# Predicting Beef Carcass Fatness Using an Image Analysis System

**DOI:** 10.3390/ani11102897

**Published:** 2021-10-05

**Authors:** José A. Mendizabal, Guillerno Ripoll, Olaia Urrutia, Kizkitza Insausti, Beatriz Soret, Ana Arana

**Affiliations:** 1 IS-FOOD Research Institute, Campus de Arrosadia, Universidad Pública de Navarra, 31006 Pamplona, Spain; olaia.urrutia@unavarra.es (O.U.); kizkitza.insausti@unavarra.es (K.I.); soret@unavarra.es (B.S.); aarana@unavarra.es (A.A.); 2 Centro de Investigación y Tecnología Agroalimentaria de Aragón (CITA), Instituto Agroalimentario de Aragón–IA2 (CITA-Universidad de Zaragoza), Avda. Montañana 930, 50059 Zaragoza, Spain; gripoll@cita-aragon.es

**Keywords:** carcass fatness, image analysis, prediction, young bulls

## Abstract

**Simple Summary:**

The degree of conformation and the degree of fatness are the primary parameters taken by the European beef carcass classification system (the SEUROP system) for assessing carcass quality and pricing. Evaluations have conventionally been performed by graders suitably trained using photographic standards but in recent years new techniques have been developed to enhance grading accuracy and objectivity. This study reports a method that uses an image analysis to assess the degree of fatness of beef carcasses. The results obtained show that the accuracy significantly improves by using this image analysis method compared with the conventional method that assigns scores based on photographic standards. It would therefore be appropriate to implement this technique on slaughter lines to improve the beef carcass classification system.

**Abstract:**

The amount and distribution of subcutaneous fat is an important factor affecting beef carcass quality. The degree of fatness is determined by visual assessments scored on a scale of five fatness levels (the SEUROP system). New technologies such as the image analysis method have been developed and applied in an effort to enhance the accuracy and objectivity of this classification system. In this study, 50 young bulls were slaughtered (570 ± 52.5 kg) and after slaughter the carcasses were weighed (360 ± 33.1 kg) and a SEUROP system fatness score assigned. A digital picture of the outer surface of the left side of the carcass was taken and the area of fat cover (fat area) was measured using an image analysis system. Commercial cutting of the carcasses was performed 24 h post-mortem. The fat trimmed away on cutting (cutting fat) was weighed. A regression analysis was carried out for the carcass cutting fat (*y*-axis) on the carcass fat area (*x*-axis) to establish the accuracy of the image analysis system. A greater accuracy was obtained by the image analysis (R^2^ = 0.72; *p* < 0.001) than from the visual fatness scores (R^2^ = 0.66; *p* < 0.001). These results show the image analysis to be more accurate than the visual assessment system for predicting beef carcass fatness.

## 1. Introduction

The EU’s Beef Carcass Classification System (SEUROP) [1] is chiefly based on two parameters: conformation and the degree of carcass fat cover. These two parameters, plus weight, are used to determine the beef carcass quality and price.

Carcass conformation and fatness are evaluated using photographic standards depicting the different conformation (SEUROP scale) and fatness (scale from 1 to 5) grades. The grades are assigned by duly accredited slaughterhouse staff trained by official agencies and who have passed the corresponding grading test. However, this method has occasionally been faulted as being subject to a certain degree of subjectivity that could result in differences in grading by slaughterhouses and even within each individual slaughterhouse depending on the grader on duty [2].

For this reason, more objective tools for classifying beef carcasses have been developed in recent years [3,4,5]. These include instrumental methods such as ultrasound [6,7,8,9], computed tomography [10,11], dual energy X-ray absorptiometry (DEXA) [12,13], a bioelectrical impedance analysis (BIA) [14], near-infrared spectroscopy [15,16], microwave systems [17] and, above all, an image analysis [18,19,20]. Artificial intelligence-based techniques for modelling carcass classification parameters by official graders have even been developed [21].

These instrumental methods achieve highly accurate measurements for one of the two main classification measures used to grade carcass quality in the EU’s classification system, namely, beef carcass conformation and have even shown to be capable of predicting commercial carcass meat yields [19,20,21,22]. However, the results for predicting carcass fatness levels do not reach the same satisfactory results because fat distribution is highly variable and not so easy to measure [19,20,23,24].

This article therefore presents an attempt to develop an image analysis application for obtaining accurate, objective measurements of the degree of carcass fatness capable of enhancing the official method based on the visual classification of beef carcasses currently in use.

## 2. Materials and Methods

### 2.1. Animals and Feeding

A total of 50 young bulls of the local Pirenaica (*n* = 25) and Asturiana (*n* = 25) breeds from northern Spain were weaned at a live weight of 271 ± 28.4 kg and 181 ± 30.2 days of age. During the feeding stage, at the CITA research center (Zaragoza, Spain), the animals were given ad libitum access to a commercial concentrate and barley straw. An initial concentrate (12.9 MJ metabolizable energy (ME) and 160 g crude protein (CP) per kg dry matter (DM)) was used up to a weight of 320 kg. Thereafter, a finishing concentrate (13.0 MJ ME and 150 g CP per kg DM) was used. The young bulls were slaughtered at 570 ± 52.5 kg live weight and 409.7 ± 27.04 days of age. Procedures involving animal care and use were conducted following the European guidelines [25].

### 2.2. Slaughter and Fatness Measurement

The slaughter of the animals was conducted at the General Refrigerated Slaughterhouse in Zaragoza according to EU regulations [26]. The hot carcass weight (HCW) was recorded after slaughter. Thereafter, the carcasses were chilled for 24 h at 4 °C and were classified using the SEUROP five-class fat cover notation system (from 15 for 5+, very high fat cover, to 1 for 1−, very low fat cover) [1]. The evaluations were carried out by two official graders.

### 2.3. Acquisition of VIA Images

**Figure 1:**
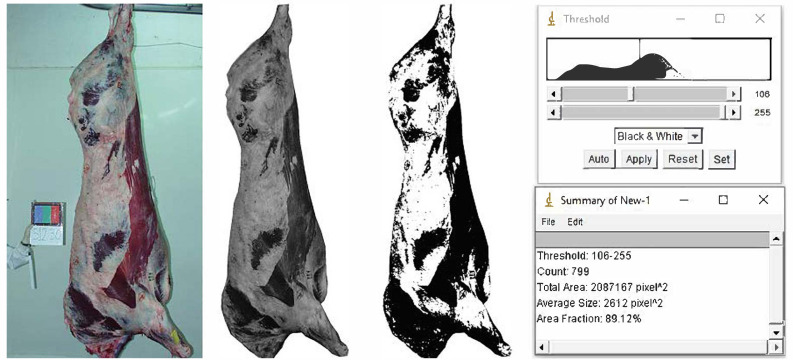
Image acquisition and processing for quantifying the carcass fat area.

A picture of each left half-carcass was taken (Figure 1) using a digital camera (Olympus E-300 SLR) with an 8 megapixel sensor (3264 × 2448) and an Olympus Zuiko 28 mm f/2.8 lens. The camera settings were: manual operation mode, aperture F/5.6, shutter 1/60, ISO 400, flash off. The camera was mounted perpendicularly to the carcass at a height of 1.75 m on a tripod placed 3 m from the carcass. The illumination intensity in the cold room where the photographs were taken was 500 lux.

### 2.4. Image Processing

The images acquired were processed using the ImageJ image analysis software (National Institutes of Health, USA). To that end, the images were binarized (8 bit format) and the contours of the area of the carcass were outlined. The carcass mean gray level was then calculated based on the gray scale, which assigns a value between 0 (pure black) and 255 (pure white) to each image pixel (gray level). The optimum gray level threshold value capable of accurately discriminating the whiter parts of the carcass (fat cover) from the darker parts (no fat cover) was then determined. The optimum threshold values for the individual beef carcasses ranged from 100 to 112 depending on the fat color and muscle color, which vary from one animal to another. It was decided to use a single threshold value for all the images in the interest of achieving a greater level of standardization for the method and, hence, the mean value of 106 was selected. Accordingly, the area obtained by grouping together all the pixels with a gray level value greater than 106 was taken as representing the carcass fat cover and given the parameter designation “carcass fat area”. Figure 1 illustrates the different steps in the method.

### 2.5. Cutting Fat

The carcasses were dressed 24 h after slaughter using the method described by Panea et al. [27] to obtain the different commercial cuts from the carcasses. During the process, the weights of the different types of fat (basically kidney and pelvic fat, intermuscular fat and subcutaneous fat) not attached to the commercial cuts were recorded and the total was designated “cutting fat”. This value was taken as an indicator of carcass fatness.

### 2.6. Statistical Analyses

An analysis of variance was used to examine the differences in the amount of cutting fat, the mean gray level and the carcass fat area values based on the fatness score assigned to the carcasses using the SEUROP system. Differences among the means were tested by Tukey’s HSD test. Simple and multiple (stepwise) linear and non-linear regression analyses were used to study the relationships among the four variables considered. The multiple regressions also included the HCW. Available variables to the models were untransformed variables; the same variables squared and base 10 and e logarithms were also tested. The third level interactions between the variables were included as an option. The variables were retained in the models when *p* < 0.05. The coefficients of determination of each model obtained (R^2^) and the standard error of the model were estimated and presented.

A statistical analysis of the data was performed using SPSS statistical software, version 27.0 [28].

## 3. Results

### 3.1. Carcass Fat Measurements

The young bull carcasses considered in this experiment were divided into four groups according to the fatness scores assigned using the SEUROP method (a scale of 1–5 with each score broken down into 3 sub-scores on a scale of 1 (low) to 15 (high)). The scores recorded were 3 (*n* = 9), 4 (*n* = 6), 5 (*n* = 28) and 6 (*n* = 7) (Table 1).

The gray level values processed from the digitized carcass images yielded mean values between 78 and 99 (on a scale of 0–255) with values increasing in line with the fatness scores (Table 1).

Finally, an image analysis quantification of the degree of fat cover of the carcasses returned a mean value of 38.4 ± 1.6% (on a scale of 1–100) with values ranging from 19.2% to 62.3%. On that basis, a fatness score of 2+, for instance, represented carcasses with a fat cover over approximately half the surface area.

### 3.2. Predicting the Cutting Fat from the SEUROP Scores

**Figure 2:**
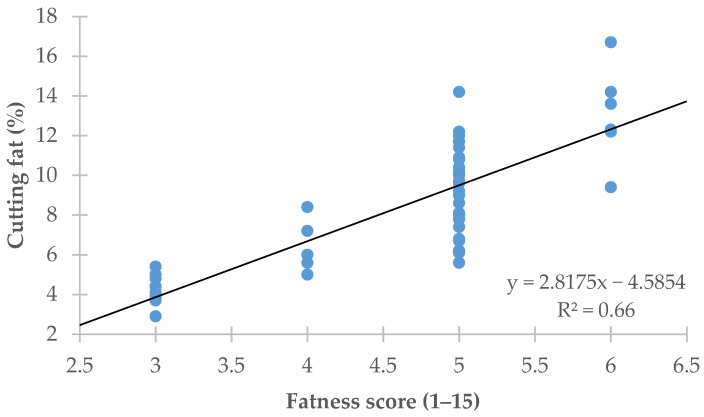
Linear regression of the cutting fat on the SEUROP fatness score.

Figure 2 plots the linear regression of the amount of carcass cutting fat (*y*-axis) on the fatness score assigned to the carcass using the SEUROP classification system (*x*-axis). The regression equation showed that the SEUROP fatness score explained 66.4% (R^2^ = 0.66) of the variation recorded in the amount of carcass cutting fat (RMSE = 1.86%).

### 3.3. Predicting the Cutting Fat from the Mean Gray Level Value

**Figure 3:**
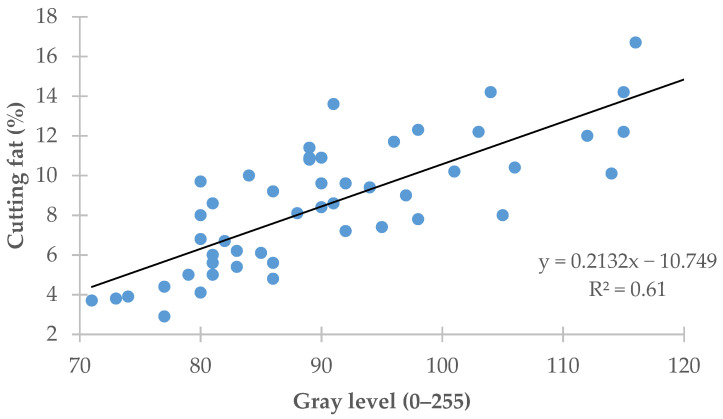
Regression of the cutting fat on the carcass gray level.

Figure 3 plots the regression of the amount of carcass cutting fat (*y*-axis) on the mean carcass gray level (*x*-axis). The regression equation showed that the mean gray level value explained 61.0% (R^2^ = 0.61) of the variation recorded in the amount of carcass cutting fat (RMSE = 2.01%).

### 3.4. Predicting the Cutting Fat from the Carcass Fat Area

**Figure 4:**
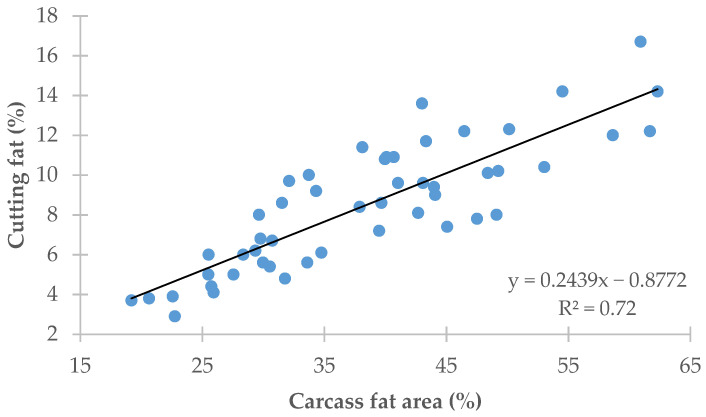
Regression of cutting fat on carcass fat area.

Figure 4 plots the regression of the amount of carcass cutting fat (*y*-axis) on the carcass fat area (*x*-axis) obtained using image analysis. Carcass fat area explained 72.0% (R^2^ = 0.72) of the variation recorded in the amount of carcass cutting fat (RMSE = 1.70%).

### 3.5. Predicting the Cutting Fat from the Set of Variables

**Figure 5:**
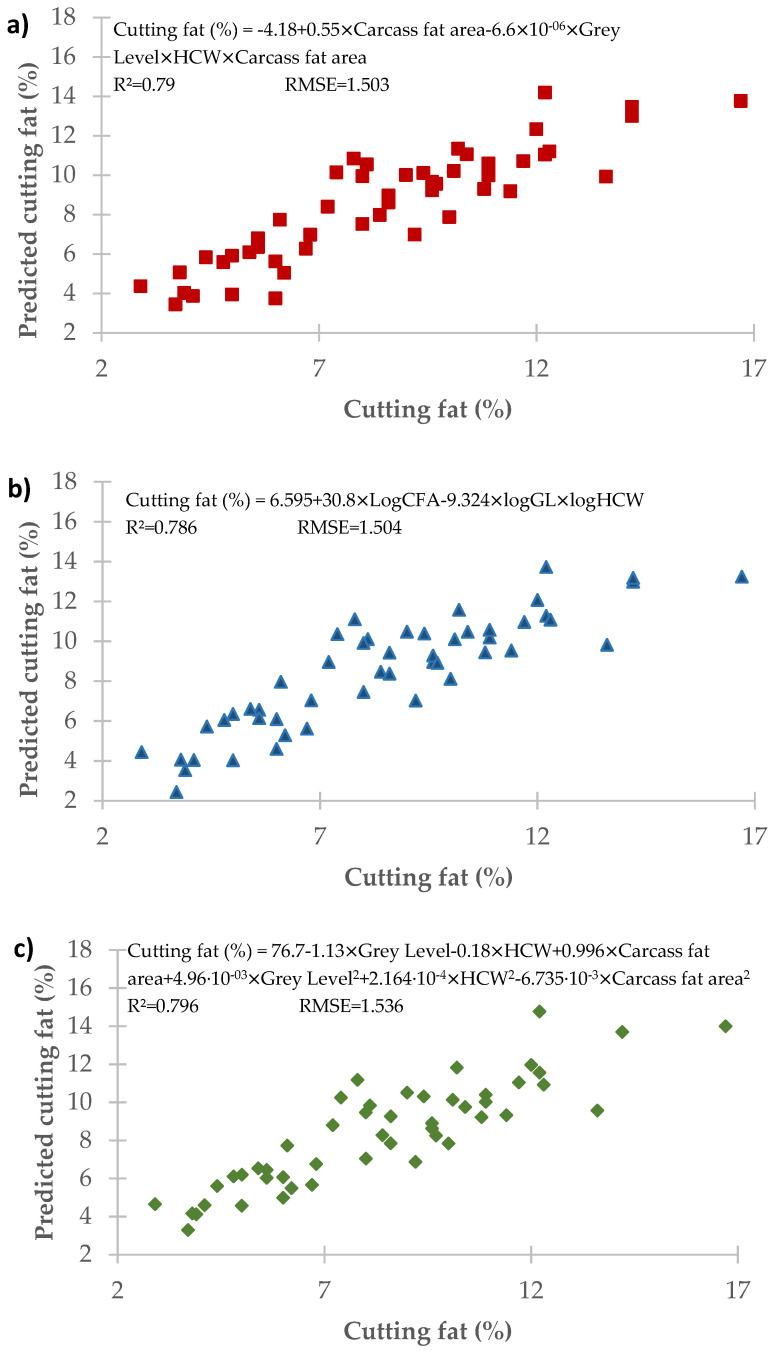
Multiple regressions. (**a**) Stepwise model based on the gray level, carcass weight and carcass fat area and their third-order interactions; (**b**) stepwise model based on the gray level, carcass weight and carcass fat area with their log transformations and fourth-order interactions; (**c**) stepwise model based on the gray level, carcass weight and carcass fat area with their log and square transformation and fourth-order interactions.

Figure 5 depicts the most salient results found on running the various multiple regressions on the set of variables considered with the HCW (hot carcass weight) variable included in the models.

These models were significantly better at predicting the carcass cutting fat, attaining values of R^2^ > 0.79 and RMSE < 1.54%.

## 4. Discussion

The EU’s SEUROP beef carcass classification system is based on assigning visual scores for carcass conformation and carcass fatness. It is widely used at slaughterhouses across Europe; however, it is often faulted for a certain degree of subjectivity in scoring by official graders and scores can vary from one slaughterhouse to another [2]. This has substantial economic repercussions as grading scores have a major impact on beef carcass prices. Albertí et al. [29] estimated that a difference of one point in a carcass’s conformation score may result in a 6 to 10% difference in the carcass’s price per kg.

This being the case, a number of different techniques are in study in an effort to make carcass grading more objective. The greatest advances in this area have possibly been made for pork. Classification systems are officially approved by EU authorities and have been implemented following the adoption by the European Commission of regulations accepting and implementing new carcass classification technologies in 2017 [30]. Work in the field on beef carcasses has been under way for several years. A number of different methods are being tested and an image analysis is one of the more advanced technologies of this kind [5,19] (Craigie et al., 2012; Allen, 2021).

One of the two assessment parameters for carcass quality in the EU’s SEUROP system, conformation assessment, has been comprehensively studied using an image analysis, obtaining very good results. The method has even been expanded to try to predict the commercial meat yields of beef carcasses, achieving very high coefficients of determination values greater than 0.80–0.90 [22].

However, less work has been performed to objectively assess the levels of carcass fatness and the results have not been as satisfactory. Fat is spread widely over beef carcasses and the distribution tends to be variable, which has been mentioned as one of the reasons for the poor results obtained up to date [19,24].

The work reported here employed an image analysis, a method that is simple yet quite objective, quantifiable and repeatable. It can provide accurate information on the degree of carcass fatness, the second of the beef carcass classification parameters. The method, as developed in this work, allows a shift from a discontinuous fatness scale of just five points (or expanded into a 15-point scale) to a continuous percentage scale from 0 to 100, which is much more comprehensive and therefore more accurate.

The results of the work reported here may have been conditioned by the type of animals included in the study. All the carcasses harvested were assigned SEUROP classification fatness scores between 3 and 6 (on a scale of 1–15) (Table 1). That is, the carcasses had little fat in spite of coming from young bulls slaughtered at weights considered high for the usual endpoint in the traditional beef production systems in Spain (570 ± 52.5 kg live weight). These fatness results were consistent with the findings reported by Albertí et al. [29] and Soret et al. [31], who described the Asturiana and Pirenaica cattle breeds as low-marbled breeds. A similar percentage cutting fat values (Table 1) were obtained for carcasses from animals of these two breeds used in the current study. This value is much less than for other faster-growing Spanish breeds such as the Avileña, Morucha or Retinta breeds, which have cutting fat values greater than 10% [29].

An image analysis provides a simple way of predicting carcass fatness by measuring the mean carcass surface area color value by way of an initial approximation. Taking the gray level value as a basis over a scale ranging from 0 (pure black) to 255 (pure white), a high gray level value for a carcass as a whole is indicative of a high level of carcass fatness whereas a low value is indicative of a very lean carcass. This can be seen in Figure 3, which shows that the predictions of the amount of cutting fat based on the mean carcass gray level attained values of R^2^ = 0.61 and RMSE = 2.01% (*p* < 0.001; Figure 3). Despite being statistically significant, these values were smaller than those obtained for the predictions of the amount of cutting fat as scored by official slaughterhouse graders based on visual assessments, which yielded values of R^2^ = 0.66 (RSME = 1.86%; *p* < 0.001; Figure 2). In other words, this method of predicting carcass fatness based on the mean carcass gray level value was not superior to the visual grading by official graders.

Carcass fat area, i.e., the difference in the gray level values for carcass fat and muscle, is a second parameter or measure for predicting carcass fatness using an image analysis. In this study, carcass scoring according to the SEUROP scale yielded values between 3 and 6 compared with carcass fat area scores ranging from 25.0% to 48.4% on the scale for this second image analysis parameter (Table 1). That is, the image analysis scale was much broader and more accurate than the scale used in the SEUROP classification system.

The accuracy achieved in predicting the carcass fatness, measured in this study as the cutting fat, was greater for the image analysis method employed than for the official SEUROP classification system (R^2^ = 0.72 vs. 0.66 and RMSE = 1.70% vs. 1.86%, respectively; Figure 2 and Figure 4).

Accordingly, the image analysis results were better than the results obtained based on the fatness score from the visual grading and yielded a more objective assessment of the degree of carcass fatness. However, the sample size used in this study was limited and it would be appropriate to increase it in future studies.

Finally, a multiple regression model that included all the variables considered in this study yielded highly accurate predictions of beef carcass fatness, attaining values of R^2^ > 0.79 and RMSE < 1.54%, showing that an image analysis is a method capable of providing quantifiable and objective predictions of beef carcass fatness.

## 5. Conclusions

The findings of this study suggest that measuring carcass fat area using an image analysis can be regarded as a suitable indicator of carcass fatness in young bulls of Spanish meat breeds. Furthermore, including this assessment method in the framework of the EU’s SEUROP classification system could be worthwhile because it provides an objective measure of carcass fatness. Nevertheless, before applying an image analysis to other breeds or production systems, the method should be tested on the carcasses of fatter animals spanning the broadest possible range of fatness scores and, if it is feasible, spanning the entire interval from 1 to 5.

## Figures and Tables

**Table 1:** Carcass fat area (mean ± se) based on the fatness scores (1–15) assigned to the carcasses.

Score (SEUROP: 1–15(1–5))	3 (1+) (*n* = 9)	4 (2−) (*n* = 6)	5 (2) (*n* = 28)	6 (2+) (*n* = 7)	*p*-Value
HCW (kg)	360 ± 12.2	358 ± 7.1	363 ± 6.8	347 ± 11.3	0.715
Cutting fat (%)	4.2 ± 0.3 ^a^	6.4 ± 0.5 ^b^	9.2 ± 0.4 ^c^	12.8 ± 0.9 ^d^	0.000
Mean gray level (0–255)	78 ± 1.6 ^a^	84 ± 2.1 ^a^	93 ± 2.1 ^b^	99 ± 3.4 ^b^	0.000
Carcass fat area (%)	25.0 ± 1.4 ^a^	31.5 ± 2.4 ^a^	41.7 ± 1.8 ^b^	48.4 ± 2.8 ^b^	0.000

Different letters (e.g., a, b, c) represent *p ≤* 0.05; the same or no letters represent *p >* 0.05.

## Data Availability

The data presented in this study are available from the corresponding author on request.

## References

[1] (1991). Council Regulation (ECC) No 1026/91 of 22 April 1991 amending Regulation (EEC) No 1208/81 determining the Community scale for the classitication of carcases of adult bovine animals. Off. J. Eur. Union.

[2] Fisher A. (2007). Beef Carcass Classification in the EU: An Historical Perspective.

[3] Cross H.R., Whittaker A.D. (1992). The role of instrument grading in a beef value-based marketing system. J. Anim. Sci..

[4] Delgado-Pando G., Allen P., Troy D.J., McDonnell C.K. (2021). Objective carcass measurement technologies: Latest developments and future trends. Trends Food Sci. Technol..

[5] Allen P. (2021). Recent developments in the objective measurement of carcass and meat quality for industrial application. Meat Sci..

[6] Griffin D.B., Savell J.W., Recio H.A., Garrett R.P., Cross H.R. (1999). Predicting carcass composition of beef cattle using ultrasound technology. J. Anim. Sci..

[7] Williams A.R. (2002). Ultrasound applications in beef cattle carcass research and management. J. Anim. Sci..

[8] Greiner S.P., Rouse G.H., Wilson D.E., Cundiff L.V., Wheeler T.L. (2003). Prediction of retail product weigth and percentage using ultrasound and carcass measurements in beef cattle. J. Anim. Sci..

[9] Beriain M.J., Insausti K., Valera M., Indurain G., Purroy A., Carr T.R., Horcada A. (2021). Effectiveness of using ultrasound readings to predict carcass traits and sensory quality in young bulls. Comput. Electron. Agric..

[10] Prieto N., Navajas E.A., Richardson R.I., Ross D.W., Hyslop J.J., Simm G., Roehe R. (2010). Predicting beef cuts composition, fatty acids and meat quality characteristics by spiral computed tomography. Meat Sci..

[11] Navajas E.A., Glasbey C.A., Fisher A.V., Ross D.W., Hyslop J.J., Richardson R.I., Simm G., Roehe R. (2010). Assessing beef carcass tissue weights using computed tomography spirals of primal cuts. Meat Sci..

[12] Lopez-Campos O., Larsen I.L., Prieto N., Juarez M., Dugan M.E.R., Aalhus J.L. (2016). Evaluation of Total Lean and Saleable Meat Yield Prediction Equations and Dual Energy X-Ray Absorptiometry for a Rapid, Non-Invasive Yield Prediction in Beef. Meat Muscle Biol..

[13] Calnan H., Williams A., Peterse J., Starling S., Cook J., Connaughton S., Gardner G.E. (2021). A prototype rapid dual energy X-ray absorptiometry (DEXA) system can predict the CT composition of beef carcases. Meat Sci..

[14] Zollinger B.L., Farrow R.L., Lawrence T.E., Latman N.S. (2010). Prediction of beef carcass salable yield and trimmable fat using bioelectrical impedance analysis. Meat Sci..

[15] Shackelford S.D., Wheeler T.L., Koohmaraie M. (2005). On-line classification of US Select beef carcasses for longissimus tenderness using visible and near-infrared reflectance spectroscopy. Meat Sci..

[16] Chapman J., Elbourne A., Truong V.K., Cozzolino D. (2020). Shining light into meat—A review on the recent advances in in vivo and carcass applications of near infrared spectroscopy. Int. J. Food Sci. Technol..

[17] Marimuthu J., Loudon K.M.W., Gardner G.E. (2021). Ultrawide band microwave system as a non-invasive technology to predict beef carcase fat depth. Meat Sci..

[18] Cross H.R., Gilliland D.A., Durland P.R., Seideman S. (1983). Beef carcass evaluation by use of a video image analysis system. J. Anim. Sci..

[19] Craigie C.R., Navajas E.A., Purchas R.W., Maltin C.A., Bünger L., Hoskin S.O., Ross D.W., Morris S.T., Roehe R. (2012). A review of the development and use of video image analysis (VIA) for beef carcass evaluation as an alternative to the current EUROP system and other subjective systems. Meat Sci..

[20] Alempijevic A., Vidal-Calleja T., Falque R., Quin P., Toohey E., Walmsley B., McPhee M. (2021). Lean meat yield estimation using a prototype 3D imaging approach. Meat Sci..

[21] Díez J., Albertí P., Ripoll G., Lahoz F., Fernández I., Olleta J.L., Panea B., Sañudo C., Bahamonde A., Goyache F. (2006). Using machine learning procedures to ascertain the influence of beef carcass profiles on carcass conformation scores. Meat Sci..

[22] Oliver A., Mendizabal J.A., Ripoll G., Albertí P., Purroy A. (2010). Predicting meat yields and commercial meat cuts from carcasses of young bulls of Spanish breeds by the SEUROP method and an image analysis system. Meat Sci..

[23] Vote D.J., Bowling M.B., Cunha B.C.N., Belk K.E., Tatum J.D., Montossi F., Smith G.C. (2009). Video image analysis as a potential grading system for Uruguayan beef carcasses. J. Anim. Sci..

[24] Heggli A., Gangsei L.E., Røe M., Alvseike O., Vinje H. (2021). Objective carcass grading for bovine animals based on carcass length. Acta Agric. Scand. A Anim. Sci..

[25] (2010). Directive 2010/63/EU of the European Parliament and of the Council of 22 September 2010 on the protection of animals used for scientific purposes. Off. J. Eur. Union.

[26] (2009). Council Regulation (EC) No 1099/2009 of 24 September 2009 on the protection of animals at the time of killing. Off. J. Eur. Union.

[27] Panea B., Ripoll G., Albertí P., Joy M., Teixeira A. (2012). Atlas of dissection of ruminant’s carcass. ITEA.

[28] IBM Corp. (2020). IBM SPSS Statistic for Windows.

[29] Albertí P., Ripoll G., Goyache F., Lahoz F., Olleta J.L., Panea B., Sañudo C. (2005). Carcass characterisation of seven Spanish beef breeds slaughtered at two commercial weights. Meat Sci..

[30] (2017). Commission Delegated Regulation (EU) 2017/1182—of 20 April 2017—Supplementing Regulation (EU) No 1308/2013 of the European Parliament and of the Council as regards the Union scales for the classification of beef, pig and sheep carcasses and as rega. Off. J. Eur. Union.

[31] Soret B., Mendizabal J.A., Arana A., Alfonso L. (2016). Expression of genes involved in adipogenesis and lipid metabolism in subcutaneous adipose tissue and longissimus muscle in low-marbled Pirenaica beef cattle. Animal.

[32] (2013). Real Decreto 53/2013, de 1 de febrero, por el que se establecen las normas básicas aplicables para la protección de los animales utilizados en experimentación y otros fines científicos, incluyendo la docencia. Off. Gaz. Spain.

